# Modelling Peri-Perceptual Brain Processes in a Deep Learning Spiking Neural Network Architecture

**DOI:** 10.1038/s41598-018-27169-8

**Published:** 2018-06-11

**Authors:** Zohreh Gholami Doborjeh, Nikola Kasabov, Maryam Gholami Doborjeh, Alexander Sumich

**Affiliations:** 1 Knowledge Engineering and Discovery Research Institute, Auckland University of Technology, AUT Tower, 7th floor, 2 Wakefield Street, Auckland, 1010 New Zealand; 2 College of Business Law & Social Sciences, School of Social Sciences, Nottingham Trent University, Nottingham, United Kingdom

## Abstract

Familiarity of marketing stimuli may affect consumer behaviour at a peri-perceptual processing level. The current study introduces a method for deep learning of electroencephalogram (EEG) data using a spiking neural network (SNN) approach that reveals the complexity of peri-perceptual processes of familiarity. The method is applied to data from 20 participants viewing familiar and unfamiliar logos. The results support the potential of SNN models as novel tools in the exploration of peri-perceptual mechanisms that respond differentially to familiar and unfamiliar stimuli. Specifically, the activation pattern of the time-locked response identified by the proposed SNN model at approximately 200 milliseconds post-stimulus suggests greater connectivity and more widespread dynamic spatio-temporal patterns for familiar than unfamiliar logos. The proposed SNN approach can be applied to study other peri-perceptual or perceptual brain processes in cognitive and computational neuroscience.

## Introduction

Neuromarketing is a relatively novel area that has been developed to understand the neurobiological mechanisms underpinning preferences towards marketing stimuli, with the view to predicting differences in consumer thought processes that might not necessarily be observable in overt behaviour^1–5^. “Branding” can be considered a major factor in consumers’ buying behaviour; thus, “brand familiarity” is typically a proxy for consumer’s preference towards marketing products^6–8^. Recent theories of response to branding distinguish sub-processes such as brand attachment and attitude towards a brand^9^, and propose that a bias towards familiar brands may occur at a very early stage in information processing.

Understanding how these early stages of processing are affected by familiarity, e.g. by logos, has important theoretical implications in the models of memory in general and applications to neuromarketing in terms of objective evaluation of product presentation and development. Consumers continuously operate with some degree of automaticity. Familiarity provokes automaticity, whilst perception and integration of features in unfamiliar stimuli require greater cognitive effort^10–13^. The majority of research on familiarity has been conducted in relation to faces, for which distinct neural mechanisms have been proposed for various types of familiarity (e.g., famous, personal and visually familiar faces^14^). Although the last three decades have witnessed development in the mechanistic understanding of subconscious behaviour in consumers^10–12,15^, models of unconscious decisions making and choices in the context of neuromarketing have not been fully delineated. Nevertheless, automaticity-related studies suggest consumers do not have access to the interior mechanisms that drive their decisions^11^. Such claims might be strengthened by expanding the currently limited empirical evidence from neurocognitive measures.

Whilst functional magnetic resonance imaging (fMRI) studies show recognised brands activate inferior frontal gyrus, anterior insula and anterior cingulate gyrus bilaterally^16^, a greater understanding of the temporal dynamics of neurocognitive processes that underpin buying behaviour might be obtained using electroencephalographic (EEG) data. EEG provides a direct measure of electrocortical activity with millisecond precision and is sensitive to changes in arousal, perception and cognitive function^17^. More specifically, EEG measures changes in extracellular potentials from large arrays of neurons, predominantly pyramidal cells. The time-locked EEG response to the presentation of a stimulus or behavioural response can be measured as an event-related potential (ERP)^18^.

Consumer-research ERP studies are often concerned with studying the late positive potential (LPP) of the ERP waveform. The LPP is a positive component that is elicited approximately 300 ms post-stimulus in response to the novel, rare or biologically salient stimuli (P3a) or during effortful target detection (P3b)^19,20^. Higher P3 amplitude could reflect an increase in resources dedicated to the direction of attention (P3a) and/or to updating memory (P3b). Neuromarketing studies utilising ERPs to investigate the post-perceptual components of ERP, such as the P300, in relation to familiarity^21^, show higher amplitudes towards familiar than unfamiliar brands, which has been interpreted as reflecting strength in categorisation and attitude towards the brand.

The high temporal resolution of ERPs makes it possible to investigate the early stages involved in cognitive processes, some of which may occur pre-consciously. Earlier components (N100, P150) occurring between 100 and 200 ms post-stimulus reflect mechanisms engaged near the onset of perception^18^. Few studies have investigated how earlier information processing stages are affected by stimuli and the related dynamic spatio-temporal patterns of brain activities^22,23^.

Prior studies have mostly focused on consumer buying behaviour in terms of directly attending to various marketing materials in their environment. However, a fundamental question is: How do marketing materials impress consumers even when they are not consciously attending to them? Observing and understanding the specific details of how these processes occur dynamically over time (especially at a subconscious level) are not investigated in depth in current neuroscience research, and little work in computational neuroscience has been performed on this topic^24^. In view of this, the current study proposes a novel computational modelling framework that is used here to develop a model of consumer behaviour that represents how early marketing materials are perceived at an unconscious level of information processing. The proposed framework is based on recent development of deep learning algorithms and neurocomputational models of spiking neural networks (SNNs) which incorporate both spatial and temporal components of data^25–35^. Various SNN architectures have been developed thus far, along with their applications for modelling and knowledge discovery across domain areas using various high-dimensional spatio-temporal datasets, including brain data^36,37^.

In this paper, a SNN-based data modelling approach is proposed for learning, modelling, visualising and a better understanding of the dynamics of neuroinformation processing and applied here on neuromarketing-related EEG data. The proposed SNN has a biologically plausible structure owing to the following reasons:

- A brain template (atlas) is used to construct a 3-dimensional SNN model that maps the location of brain structures.
- Spatial mapping of input features (data variables) in the SNN model preserves spatial information in the brain data.
- Input data are encoded to spikes, emphasising certain changes in the brain data (signals) at a millisecond time scale.
- Initialisation of the SNN model uses the brain-inspired small-world connectivity rule.
- Biologically plausible learning rules are applied to evolve the SNN functional connectivity in a deep learning mode, resulting in long chains of connections.

In the current study, we demonstrate for the first time that such SNN models can learn deep spatio-temporal patterns of EEG/ERP data, reflecting peri-perceptual processes during a neuromarketing experiment in which familiar and unfamiliar logos are presented. The proposed SNN architecture reveals unstudied components of perception of familiar and unfamiliar brands at a peri-perceptual level. This SNN architecture provides unique, novel insight into a window of neurocognitive processing that has essentially been technically infeasible thus far.

## Results

### Statistical Analysis of ERPs

**Figure 1:**
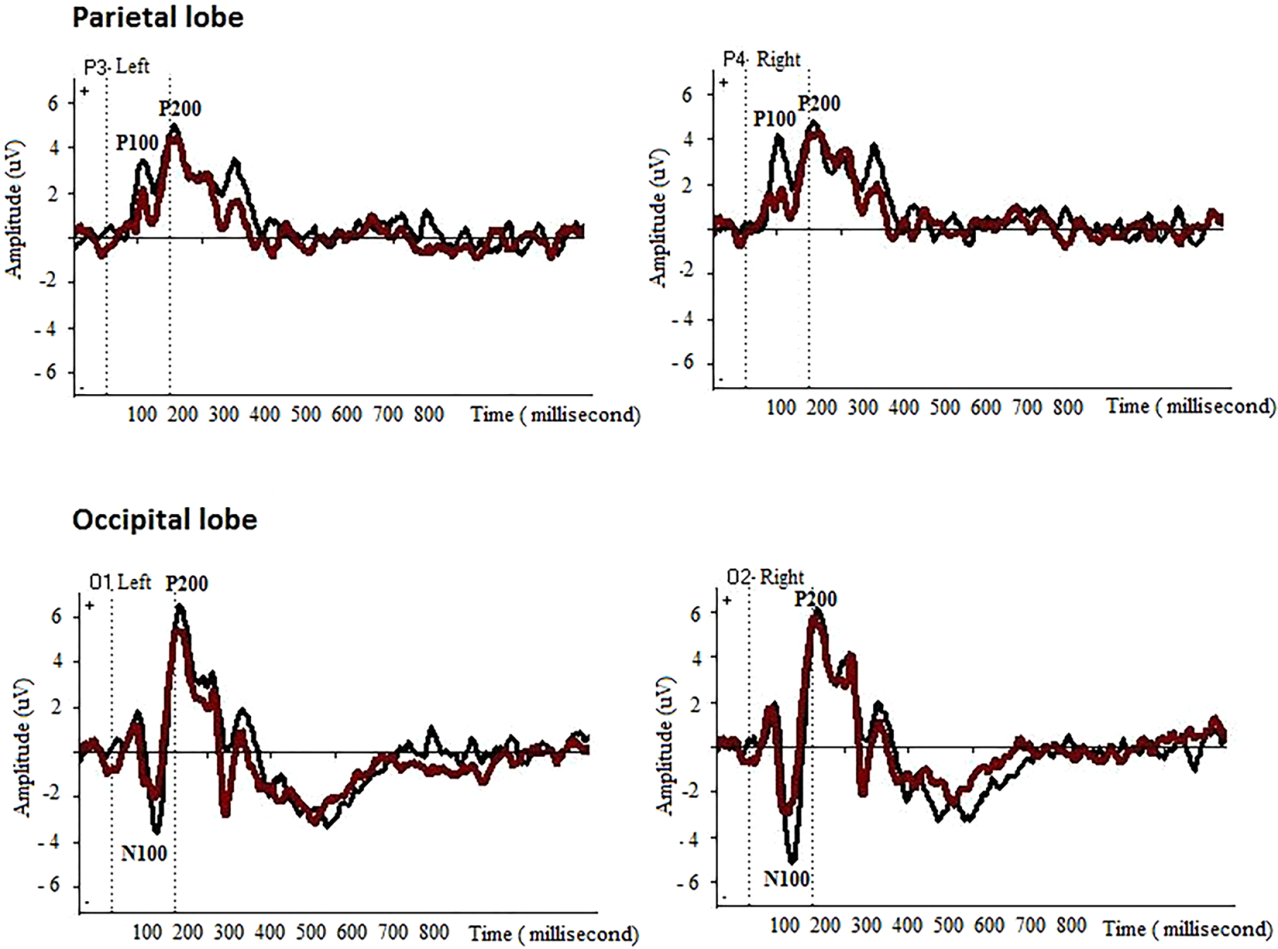
Grand average ERP waveforms of 20 subjects across 800-milliseconds epoch after familiar versus unfamiliar marketing stimuli presentation. Peak amplitudes correspond to the P100, N100 and P200 components of ERPs across the EEG channels located at posterior areas of the brain (O1, O2, P3 and P4 channels). Black line = familiar logos; red line = unfamiliar logos.

As an initial analysis, WinEEG (Mitsar system) was used to derive grand-averaged ERP waveforms across 19 channels. Analysis of ERPs was confined to occipital and parietal electrodes – O1, O2, P3 and P4 – where the peak amplitudes for early ERP components (N100 and P200) were maximal compared to other sites, in the task currently used (Fig. 1).

Mean amplitudes of early components of ERPs in posterior regions were extracted (P100, 100–180; N100, 100–180 ms; and P200, 180–240 ms) (in Supplementary Tables 1 and 2). The mean amplitudes of P100 in Parietal lobe and N100 in Occipital lobe were higher in the right hemisphere for familiar logos than for unfamiliar ones. Analysis of variance (ANOVA) was used to test for difference between variables (ANOVA)^38^ are reported in Supplementary Tables 3 and 4.

Repeated-measures ANOVA was performed separately for each time window, with respect to Familiarity (familiar, unfamiliar) and Electrode Site (right, left) in the occipital and parietal lobes for 20 subjects. For the parietal P200, there was a significant main effect of the factor Familiarity [F (1, 18) = 4.54, p = 0.04 and a significant Electrode Site*Familiarity interaction [F (1, 18) = 4.61, p = 0.01].

For the occipital N100, there were significant main effects of Electrode Site [F (1, 18) = 11.45, p = 0.01)] and Familiarity [F (1, 18) = 3.51, p = 0.01]. A significant interaction between Electrode Site and Familiarity was also observed [F (1, 18) = 4.66, p = 0.04)].

### ERP Data Modelling with the SNN-based Methodology

The brain is a highly interactive and deep learning network, but nearly all multivariate models employed in cognitive neuroscience are linear and do not model interactions. Understanding of the dynamic patterns of spatio-temporal brain data through the above traditional analysis is limited because temporal features manifest complex interactions that change dynamically over time. Therefore, it is crucial to develop new computational models that are capable of learning spatio-temporal interactions between multivariate data streams. SNNs are the third generation of neural networks and comparing to conventional neuronal networks which deal with static vector-based data (temporal information needs to be converted into vectors of static features)^33^, SNNs incorporate spatio and temporal components of data into operating. One of the significant aspects of SNNs is their compact representation of space and time that makes them suitable for learning spatio-temporal brain data (STBD) and for their analysis, where spatio and temporal information are both essential to be preserved.

Therefore, SNN is a way of using spike-time dynamics to extract interactive structures from the brain data, without over-fitting to a particular classification problem, and which constrains the immense space of possible interactions in a biologically plausible way.

We hypothesise that a properly designed SNN model can be used to model brain data and to detect deep spatio-temporal patterns for a better understanding of data. In the second phase of analysis, we have applied the SNN-based methodology to evaluate how peri-perceptual processes of the brain can be modelled and understood. Specifically, spatial and temporal features of EEG data are modelled together to better understand the interactions and relationship between the data variables over time.

**Figure 2:**
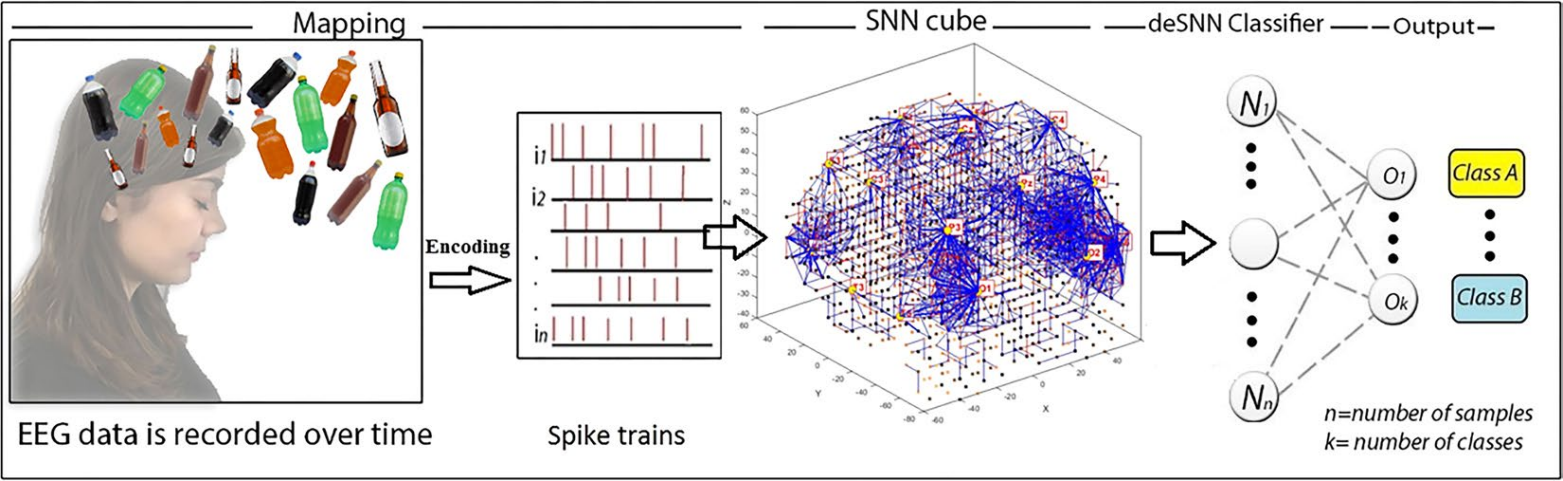
The proposed SNN architecture performs as follows: encoding EEG data as spike sequences; mapping of these sequences into a 3D SNN model created with the use of a brain template; unsupervised learning of the spike sequences in the SNN model; supervised learning and classification of the learned patterns in a SNN classifier. The Figure was drawn by authors Z.G. and M.D.

The proposed SNN architecture includes the following functional modules (shown graphically in Fig. 2):

1. **Mapping**: Spatially map EEG data into a 3D SNN model that represents a brain template;
2. **Learning**: Train the SNN model using spike-time learning rules with the EEG epochs extracted within 50–200 milliseconds post-stimulus time window;
3. **Pattern visualisation**: Visualise the deep-learned patterns of interactions between the EEG channels over time as evolved chains of connectivity in the SNN model;
4. **Classification**: Classify the learned patterns of spiking activity when familiar and unfamiliar logos are presented.

To investigate the consumer performance towards familiar-related logos versus unfamiliar-related logos, ERP time series of different time intervals (50, 100, 150 and 200 ms after stimuli presentation) related to the peri-perpetual processes of the brain were used. EEG data were mapped using the Talairach brain template^39,40^, as it was also the basis for the 3D SNN model, defining the positioning of the spiking *neurons* in the model. The mapped SNN models were initialised using the small-world connectivity rule^41,42^ in which a probability of a neuron to be connected to another neuron depends on the distance between the two neurons, the larger the distance – the smaller the probability. To speed up this initialisation, a radius that defines the maximum distance of connections of one neuron to another in the 3D space of the SNN can be defined. We assigned a radius equals to 2 which means two neurons away from each direction in (x,y,z) coordinate in the 3D SNN model. The initial connections are assigned with small random weights, so that on average 80% of them are weighted by positive values while 20% of them are weighted by negative values, uniformly distributed, as commonly used in such studies (see^43,44^).

**Figure 3:**
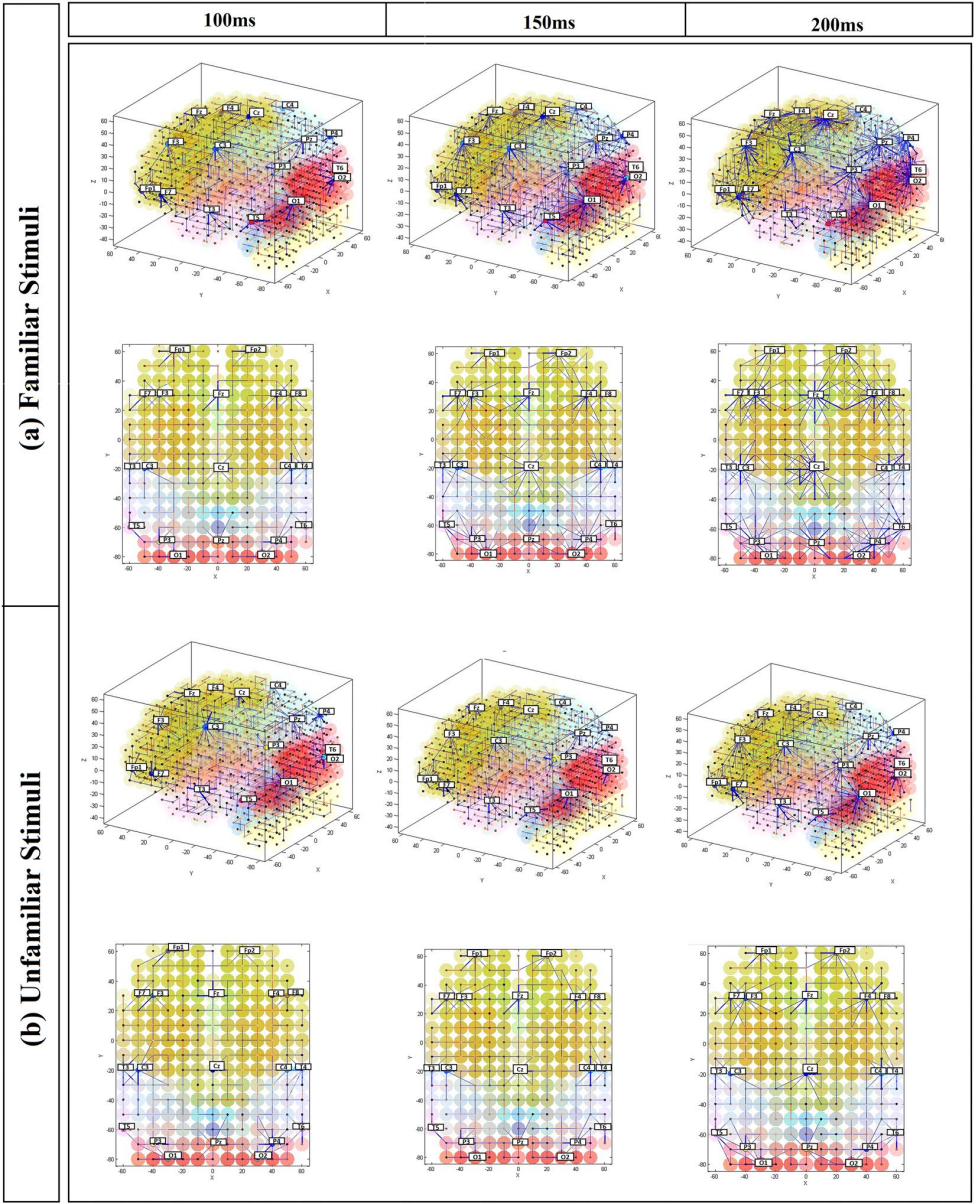
Neuronal connections created during learning in the SNN models, reflecting the dynamic patterns of EEG data corresponding to different epoch lengths: 100 ms, 150 ms and 200 ms after presentation of (**a**) familiar marketing logos and (**b**) unfamiliar ones. Excitatory connections are represented by blue lines, while inhibitory ones are in red. The thicker the line, the greater the enhancement of the connection captured after the learning process. Neurons in the SNN models are labelled by eight brain areas from the Talairach template^39,40^: Temporal (pink), Parietal (light-blue), Frontal (yellow-green), Sub-lobar (orange), Cerebellar (light yellow), Limbic (green), Pituitary (blue) and Occipital (red). The connection weights are averaged and reported for each SNN model. For a clear visualisation, we only visualised the connection weight greater than 0.08. The pictures show that familiar stimuli result in a higher connectivity and higher connection weights at average.

To train the SNN model, EEG signals are first encoded as sequences of binary events of 1 and −1, called spikes, representing the positive and negative changes, respectively, in the signal time series. A threshold-based representation (TBR)^45^ technique was applied to every EEG channel time series to encode it as a spike sequence. Figure 3 shows a spatial mapping of EEG electrodes into the same 3D space of spiking *neurons*, positioned according to the Talairach template^39,40^. The generated spike trains from EEG channels are then entered into the specially mapped SNN models via input neurons and the spatio-temporal patterns of EEG data were captured in the form of neuronal connectivity. In Fig. 3(a,b), we visualise the neuronal connections created during the Spike Time-Dependent Plasticity (STDP) learning in the SNN models, reflecting the dynamic patterns of EEG data corresponding to different epoch lengths: 100 ms, 150 ms and 200 ms after presentation of familiar and unfamiliar marketing logos. The average weight of all neuronal connections in each SNN model is also reported in Fig. 3 as a metric for comparison. In Table 5 of the Supplementary Material provided, we report the average weight of the neuronal connections that were formed around each EEG channel (between input neurons and its connected neurons). The connections, generated during learning for an input neuron, reflect on the changes of the data in the corresponding EEG channel. As many input neurons spike at different times, reflecting on the dynamics of brain activity, clusters of neurons get connected in a chain, reflecting on the temporal dynamics in the multivariable brain data. The SNN creates a functional connectivity model, where many-to-many neurons become connected to capture functional dynamical patterns from the data, even though the learning rule is applied to neuron-to-neuron connections.

**Figure 4:**
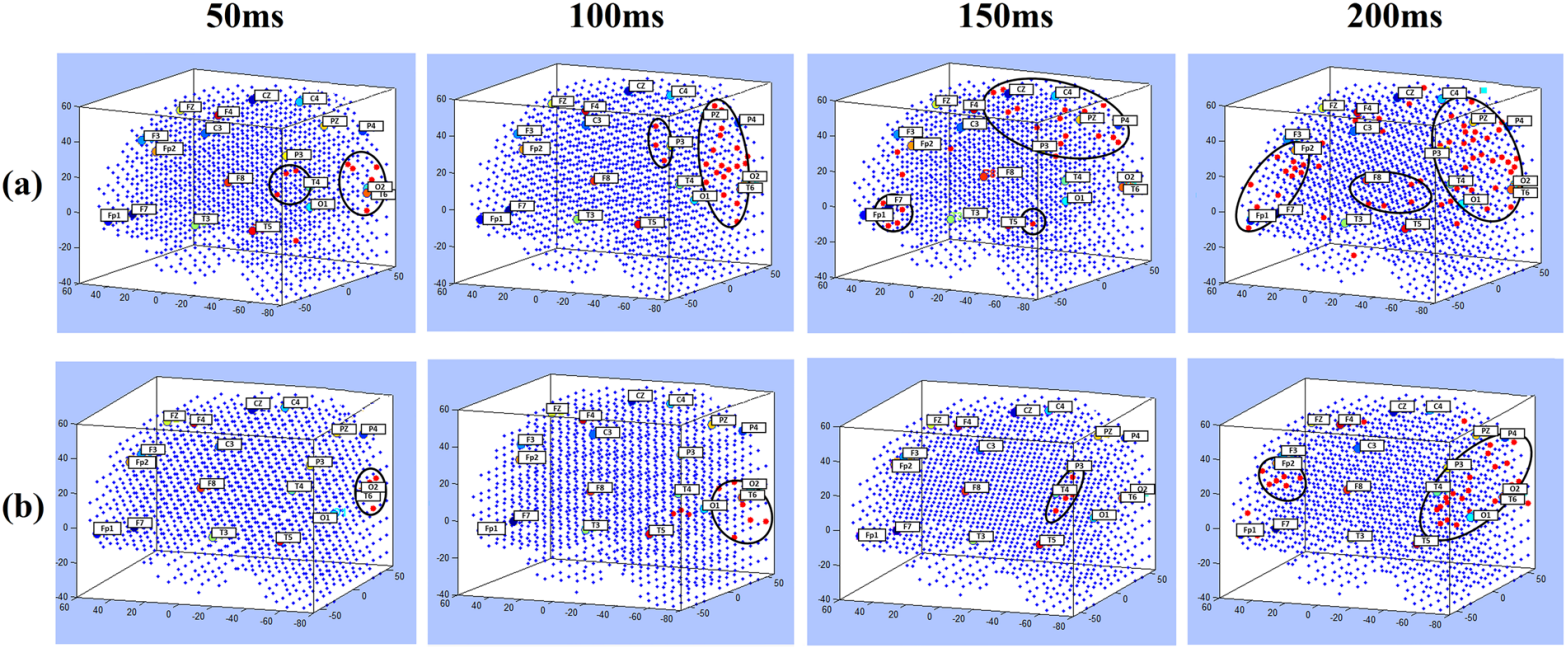
Clusters of active neurons (spiking) in the 3D SNN models are illustrated for every 50 ms while learning from the input EEG data streams of (**a**) familiar logos and (**b**) unfamiliar ones. The value A refers to the number of active neurons at each time frame.

During the STDP learning process in SNN models, consecutive snapshots of the firing state of the neurons were captured to represent a trajectory of dynamic, deep-learned patterns of neurons’ spiking activity with respect to the temporal order in which clusters of neurons emitted spikes. Figure 4a,b illustrate the sequential spiking activity patterns in the SNN models for familiar and unfamiliar logos. The earlier a cluster of neurons (surrounding an EEG channel) fires in time (shown as red neurons, which their post-synaptic potential crosses the firing threshold and emits an output spike), the earlier spiking activity is observed in a chain of functional activity. It illustrates how early different areas of neurons in the SNN models fired (sent their spikes out) at different time frames (every 50 ms) towards familiar and unfamiliar logos. Although there was a similar pathway of spiking activity in both models, the size of the activated clusters of *neurons* was significantly different between the familiar logos and the unfamiliar ones. Numerical information about the number of spikes in time and space is also reported in Fig. 4. A comparison between the activated neurons in SNN models, shown in Fig. 4, is presented in Supplementary Table 6.

**Figure 5:**
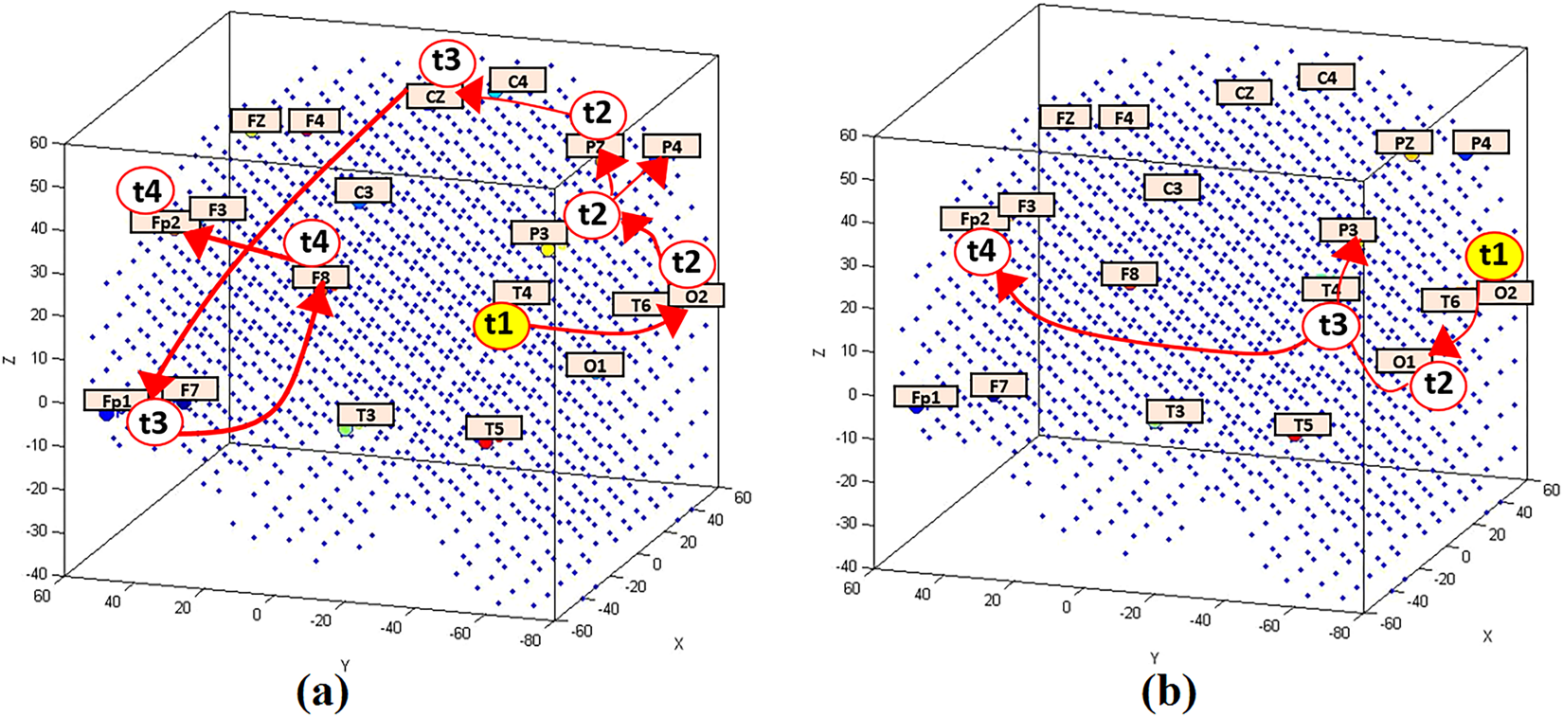
Spatio-temporal patterns of activities in the trained SNN models shown as trajectories of 4 aggregated stages (t1 = 50, t2 = 100, t3 = 150 and t4 = 200 ms) during learning in the SNN models for (**a**) familiar logos versus (**b**) unfamiliar logos. In fact, the time for a deep-learning step in the SNN model is a millisecond, and the actual activation trajectories (chains) in the SNN are 200 neuronal clusters long, but here the activity of only 4 steps of learning are visualised.

Considering the temporal order in which clusters of neurons around the EEG channels emitted spikes (red neurons) during 200 ms (one frame every 50 ms), we captured a chain of sequentially activated areas as a trajectory of deep-learned patterns in the SNN models. As illustrated in Fig. 5, the trained SNN model forms a deep architecture as whole spiking input sequences which are learned as chains of spiking activities. Unlike hand-crafted layers used in second-generation neural networks^46–50^, or randomly connected neurons in the computing reservoir of a liquid state machines^32^, the chains of directional connections established in our proposed SNN model (Fig. 5) represent the spatio-temporal relationships (adapted over time) between the sources of the spike sequences (the input variables). Due to the scalable size of a SNN model, the chains of connected neurons are not restricted in length during learning, which can be considered as unrestricted deep learning, in contrast to existing deep learning methods that use a fixed number of layers.

During the STDP learning process, we measured the intensity of the spikes in a cluster of neurons around each EEG channel. The intensity is measured as a percentage of the number of neurons that fired among all the neurons that are connected to an EEG channel. The spike intensity is reported in Supplementary Table 7. In Table 1, the activated areas in the SNN models are labelled as low, medium and strong levels of activation with respect to the number of spiking *neurons* involved at each time frame. As defined in Supplementary Fig. 5, the activation level is categorised to low (from 0.1 to 0.4) medium (from 0.4 to 0.7) and high (from 0.7 to 1) representing the percentage of the fired neurons among all the connected neurons to an input neuron. The spike intensity in a cluster of neurons around each EEG channel *i* is defined as a percentage of the number of fired neurons divided by the number of connected neurons to *i*. The maximum level of activation at each time frame *t* is 1 which means all the connected neurons to *i* fire, while 0 refers to the minimum level of activation which means no neuron fires. We partitioned this interval to three levels as shown in Supplementary Fig. 5.

**Table 1:** The activated brain areas are reported according to the numbers of activated *neurons* in the SNN models during learning, over time steps: 50, 100, 150 and 200 ms.

Classes	Brain areas involved at different time frames				
	Activity intensity	50 ms	100 ms	150 ms	200 ms
Familiar	Low	T4	P3	F7, Fp1	O1, F7, Fp1
	Medium	O2	Pz, O2, T6	Pz, P3, P4,Cz	F8
	Strong	—	—	—	O2, T4, Pz, P4, Fp2
Un-familiar	Low	O2	O1, O2, T6	P3, T4	O2, T6
	Medium	—	—	—	O1, P4
	Strong	—	—	—	T4, Fp2

The level of activation is denoted as low, medium and strong. To perform a comparative analysis, we used conventional methods Multilayer Perceptron (MLP), Multiple Linear Regression (MLR) and Support Vector Machine (SVM) for classification of EEG data as reported in Table 3. The classification problem was performed using leave-one-out cross validation.

This confirms the clear discrimination between perceptions of familiar versus unfamiliar logos at the subconscious level.

When the unsupervised training of the SNN models was completed, we applied a SNN supervised learning algorithm^35^ to train a classifier to identify whether SNN model activity was generated in response to the familiar or unfamiliar logo. As described in the materials and methods, twenty participants each performed a three-block cognitive task that involved the presentation of both familiar and unfamiliar stimuli. Therefore, for each participant, there are three EEG samples per class (6 samples per participant). In total, we had 120 EEG data samples used for the classification problem using a leave-one-out cross-validation method. This method involves the creation of 120 models, one for each sample of data, training the model using the remaining 119 samples, and testing the accuracy of each of these models for the left-out sample (unseen sample). For optimisation, we performed an exhaustive grid search on the combination of parameters for every model. Each parameter was searched within a range, specified by the minimum and maximum, through a number of iterations related to the number of steps for moving from minimum to maximum. For every model created out of 120 models, we chose three main parameters (STDP learning rate, neuron firing threshold, classifier parameter *mod*) to be optimised. The parameters were selected by assigning 10 steps between the minimum and maximum values of each parameter. Therefore, for every model creation, 1000 iterations of training (using 119 samples) and testing (using the single holdout sample) were performed using a different combination of these three parameters. Then the parameters that resulted the best accuracy in most of the iterations, have been reported as the optimal parameters. When the optimisation procedure was completed, the most selected values for the parameters across all the 120 models were selected as: STDP learning rate = 0.01; neuron firing threshold = 0.5; deSNN classifier parameter *mod* = 0.4. Table 2 presents the overall classification accuracy for the two classes of stimuli.

**Table 2:** The classification accuracy of 120 EEG samples of familiar logos (class 1) and unfamiliar logos (class 2) are obtained using leave-one-out cross validation in a SNN model.

The proposed SNN-based methodology						
Predicted	Familiar Stimuli (C1)	Unfamiliar Stimuli (C2)	Total accuracy %	F-Score %	Sensitivity %	Specificity %
Real						
Familiar Stimuli (class C1)	**52**	8	**83.00**	84.00	84.00	86.00
Unfamiliar Stimuli (class C2)	10	**50**				

In the confusion table, the rows are the real values and the columns are the predicted values.

## Discussion

This paper proposes a new methodology and a SNN model for training on EEG data to capture differences in dynamic brain activation patterns corresponding to peri-perceptual processes in response to familiar and unfamiliar stimuli, exemplified here as marketing logos. The proposed SNN architecture reveals unstudied components of perception of familiar and unfamiliar stimuli at a peri-perceptual level. This SNN architecture provides unique, novel insight into a window of neurocognitive processing that has essentially been technically infeasible thus far. When compared with traditional machine-learning techniques or deep-learning neural networks^25–31^, the proposed SNN model has the following advantages: (1) it preserves the spatio and temporal information both together in one model and can be interpreted as this model is spatially structured according to a brain template. (2) It does not have a fixed structure or number of layers, so can be as deep as required according to data size. (3) It learns spatio-temporal patterns from data through biologically plausible learning rules. (4) It allows for fast, on-line learning. (5) It allows for interpretation of the interactions and relationships between the brain data variables as reported in Table 1 and Fig. 5. (6) It offers a better classification accuracy compared to conventional methods, as reported in Table 2. The classification results of EEG patterns learned in a SNN model confirm that the model can discriminate with a high accuracy spatio-temporal patterns generated by familiar versus unfamiliar stimuli at an early stage of cognitive processing (around 200 ms).

As illustrated in Fig. 1 and Supplementary Tables 1 and 2, the ERP analysis indicates that familiar items are associated with larger response amplitudes over the posterior regions. This might mean that both kinds of stimuli drive activity over the same regions, but familiar items drive more activity. Beyond the ERP results, the proposed SNN models discovered the differences in the scalp areas involvement between familiar and unfamiliar logos at different time points. It means that SNN models can learn and identify which areas of the brain contribute to an increase in ERP and also - how does it happen over time. We could not draw such a conclusion from the ERP analysis only. In the current study, the SNN-based methodology is used in integrating the temporal and scalp topographic information, such that we obtain a better understanding of the pathways of information processing, in addition to have discrete measurements of neuronal response (e.g. ERP component amplitudes).

The SNN models trained on familiar logos suggest stronger connections (Fig. 3), even at early processing stages (e.g., 200 ms), across the EEG channels compared to the SNN models for unfamiliar logos. For unfamiliar logos (Fig. 3b), connections are generally uniform and cannot be differentiated between the channels at any processing stage in the SNN model.

Given that perceptual speed increases as individuals adapt to features of the environment, the current findings might reflect a more rapid spread of activation in response to familiar stimuli because they are more commonplace in individuals’ environments. Alternatively, it could be that certain characteristic features of the familiar stimuli lead to greater activation across brain regions through activation of schemas (those cognitive frameworks or concepts that used for organising and perceiving new information)^51^. Consumers pay more attention to the stimuli that assimilate into their schema while re-interpreting conflicts to the schema as exceptions or reshaping them to assimilate^52^. Indeed, consumers continuously operate with some degree of automaticity. The more familiar the stimuli, the more routine the behavior^53^. In contrast, the more novel the stimuli or environment, the more the conscious mind attends to the circumstances^54^. Whilst these two hypotheses are not mutually exclusive, their differentiation would require further support experimentally in relation to the SNN output as a function of familiarity schema activation.

Whilst differences in spatio-temporal activation patterns were most prominent when data from the 200 ms epoch were streamed for the training process, more subtle differences were observed at earlier time points, supporting the SNN models as being able to distinguish brands of varying familiarity in brain activation patterns at a peri-perceptual stage. For example, as shown in Fig. 4 at the 50 ms time point (T1), activation is observed over occipital and temporal regions for familiar logos but is restricted to occipital sites for unfamiliar stimuli. This may reflect top-down input to perception of temporal regions, for example from those governing memory and or emotion^55^ for familiar logos. Activation for familiar logos then takes more widespread parallel dorsal and ventral routes to activating frontal regions, with possible feedback loops to occipital cortex. Whether these routes relate to the “where/how” (dorsal) and “what” (ventral) pathways for visual perception^56^ should be investigated in future research. Such work would provide insight into whether greater restriction of the response to unfamiliar logos to the ventral pathway is due to a primary goal of the observer in object recognition (i.e., processing “what” the object is). In comparison, larger semantic networks may be activated in response to the familiar logo.

Experimental results are illustrated here mainly to represent visual exploration of the SNN models, but numerical information (such as connection weights and spiking intensity) are also facilitated and can be exported from the models. For comparative analysis we calculated the average value of connection weights in each trained SNN model (Fig. 3) and reported this number as activation level towards each stimulus (Familiar and unfamiliar). We obtained a higher activation level of 1.01 in the trained SNN model that corresponds to familiar stimuli at 200 ms post-stimulus (see Fig. 3, connection weights). Our findings suggest that stronger functional connectivity may indicate increased interplay of activated brain areas underlying cognitive functions. More information can be obtained from the Supplementary Table 5 in which the averaged connection weights for every single EEG channel are reported for both familiar and unfamiliar stimuli. It shows a higher average of connection weights towards familiar stimuli at every time frame (0.35, 0.70 and 1.01 at 100, 150 and 200 ms respectively).

Figure 4 illustrates the sequential spikes in the SNN models for familiar and unfamiliar logos from 50 ms to 200 ms. This figure is supported by numerical information which represents the number of spikes emitted at each time frame. It shows more neurons fired and sent out spikes in the SNN model of familiar than unfamiliar in all time frames. This information is also reported in Supplementary Table 6 that shows for instance, the intensity of spike activation for familiar is 3.7 times greater than unfamiliar. In order to interpret which EEG channels were mostly involved in the spiking activity at each frame, Supplementary Table 7 was presented with respect to the intensity of activation measured for each EEG channel. This intensity was computed with respect to the percentage of the number of spikes in a cluster of neurons around an input neuron (connected neurons to input neuron). It shows that the Pz, P4, T4, Fp2 and O2 channels at 200 ms post-stimulus had greater intensity of spikes emitted during the learning process for familiar stimuli than unfamiliar ones.

Table 2 summarises the classification accuracy achieved from the proposed SNN method while Table 3 represents the results of conventional learning techniques. In Table 2 a confusion table is reported to show the miss-classified samples versus the correctly classified ones. The conventional machine learning methods presented in Table 3 deal with vector-based data and do not model the spatio-temporal interactions related to the processes that generated the data as it is in the SNN models. Table 2 shows that applying SNN for classification of spatio-temporal data resulted in significantly higher accuracy as compared with conventional methods such as MLP, MLR and SVM. This can be justified with respect to a vital aspect of SNN that can preserve time information along with the spatial information of the sources of temporal data. In the proposed SNN model, each data sample for training and for testing the model represents the intensity of all EEG channels within a whole time interval, e.g. 200 m sec. During the training process, the temporal information of all channels is entered as a data stream to the SNN model through the spatially mapped input neurons and the spiking neurons were dynamically processing these inputs. However, in the conventional machine learning methods, each sample is a single input vector, where neither temporal- nor spatial information of the data is adequately represented.

**Table 3:** The classification accuracy of EEG data using Multilayer Perceptron (MLP), Multiple Linear Regression (MLR) and Support Vector Machine (SVM) through leave-one-out cross validation (computed using NeuCom at www.theneucom.com).

Traditional Machine Learning Methods			
Methods	MLP (Multi-Layer Perceptron)	MLR (Multiple Linear Regression)	SVM (Support Vector Machine)
Accuracy in %	47.50	37.50	37.50
F-Score	40.00	39.50	41.00

The MLP configuration is: Number of Hidden Units: 9; Number of Training Cycles: 1800; Output Value Precision: 0.0001; Output Function Precision: 0.0001; Output Activation Function: linear. The SVM configuration is: SVM kernel: Polynomial, Degree Gamma: 1.

Thus, the current results illustrate that our proposed methodology is promising and suitable for pattern recognition of peri-perceptual brain activity in response to stimuli familiarity. Future work will investigate whether it could be used as a tool in the early detection of spatio-temporal patterns generated by other stimuli in relation to neuromarketing. For example, the model might be further developed to recognise patterns of choice behaviour, and as such could be used to direct marketing strategies. Thus, future studies should evaluate the potential for refinement of the model and application of peri-perceptual processing measures to neuromarketing, as an objective measure of consumer preference for logos and product presentation. Although the present study has investigated familiarity to logos, the current findings might not be restricted to such stimuli. Thus, future research should investigate whether similar effects are seen in relation to familiarity to other stimuli, for example, faces.

Other studies have investigated the roles of the social environment, social attributes and the reward system in choosing familiar brands^57,58^. Thus, future work should investigate brain activation patterns using the SNN model in terms of psychosocial factors, personality variables and inter-individual differences that may affect a person’s cognitive response to familiar logos and, indeed, brand preference. For example, future studies should investigate the early and late ERP components in relation to brand attachment and brand attitude. One might expect the earlier components to relate to an empathic attachment to the brand.

In summary, the results of previous neuromarketing research suggest that frontal regions are widely engaged in consumers’ preferences and attentional functioning. The results of our study confirm these findings, but further extend it to suggest that this effect can be deeply understood through evaluating the activation time and spiking intensity across peri-perceptual regions affected by marketing stimuli. Making use of a SNN based methodology enabled us to have a better understanding in terms of the dynamics of the brain processes under performing a complex cognitive task. The proposed SNN approach can be applied to study other peri-perceptual brain processes, such as processes related to decision making, to human past-experience and/or to human preference.

## Materials and Methods

The research is supported by the Knowledge Engineering and Discovery Research Institute at Auckland University of Technology (www.kedri.aut.ac.nz), and all experiments were performed in accordance with relevant guidelines and regulations.

### Participants

Twenty right-handed volunteers, who had no neurological abnormalities, participated in the data acquisition procedure (10 males with mean age of 24.40 and Standard Deviation = 1.33; 10 females with mean age of 22.60 and Standard Deviation = 2.87). The recording procedure was performed in the “Hamrah Clinic” of Tabriz, Iran. The EEG data were recorded from human participants. Prior to commencing this research, ethical approval was granted by the “Ethics Committee of the Hamrah Clinic, Tabriz, Iran”, and informed consent was provided by all participants. Identifying information of participants, including names and initials, is not reported in the written descriptions.

### Cognitive Task Description

Prior to completing the task, participants listened to a short story about choosing a drink brand, in order to equalise the participants’ context and engage attention to the paradigm. Participants completed a visual oddball paradigm^59,60^ that consisted of three blocks. Every block started with the target logo presentation (a logo for water) that was presented 28 times in each block randomly (pre-set order) dispersed among 8 non-target logos (4 locally widely familiar logos and 4 non-familiar logos), each presented 14 times. Thus, 140 stimuli (duration = 200 ms; inter stimulus interval = 1300–1500 ms) were presented in each block. Participants were instructed to respond to the target logo as soon as they observed it on the screen (counterbalanced across participants to press either the left or right mouse button with left or right hand). In this task, the same target stimulus was used for all the subjects. Prior to designing the cognitive task, the brand familiarity was measured using a survey that was done in various local supermarkets in the city where the EEG data were recorded. Therefore, we have collected comprehensive information in terms of well-known/unknown brands, brands that were frequently requested by consumers in the supermarkets, and other measures. Therefore, through this survey, we could select the locally most familiar and unfamiliar brands as our stimuli.

### Electrophysiological Acquisition

EEG was measured through nineteen channels: C3, CZ, C4, F7, F3, FZ, F4, F8, P3, PZ, P4, T3, T4, T5, T6, FP1, FP2, O1, and O2 positioned in an adaptable cap with the standard 10–20 configuration^61–63^. The EEG data sampling rate was 256 Hz. The channel Fpz was used as a ground electrode, and all the channels’ impedances were under 5 kΩ. Off-line artefact rejection was used to eliminate the effects of eye/muscle movements. To run the cognitive task on a PC monitor, Psytask software was used as a stimulus presentation system. During the task presentation, event-related potentials (ERPs) were also measured along with EEG data.

### The SNN Architecture for Analysis of Spatio-Temporal Brain Data

Spiking neural networks (SNNs) are computational models that are inspired by the brain’s neuronal structure. In a SNN, an artificial spiking *neuron* is an information-processing unit that learns from input temporal data over time to resemble the learning processes of the brain. Spiking *neurons* are interconnected through their synapses, which memorise the learning patterns. They incorporate the concept of time into their operating models. SNN models have improved the level of biological plausibility in neural networks. Therefore, SNNs are considered suitable models for processing spatio-temporal brain data (STBD). A SNN model can be implemented using several models, such as leaky integrate-and-fire (LIF) models (as shown in Supplementary Fig. 1). In this type of model, the post-synaptic potential (PSP) of a spiking *neuron* increases or decreases with respect to every spike from a pre-synaptic *neuron*, modulated by the corresponding synaptic connection weight. As soon as the PSP reaches a threshold θ, the *neuron* generates an output spike and sends it to its connected neighbours. Its PSP then resets to a baseline value. The PSP can leak by a certain value (temporal parameter τ) when no spike arrives within a given time period.

**Figure 6:**
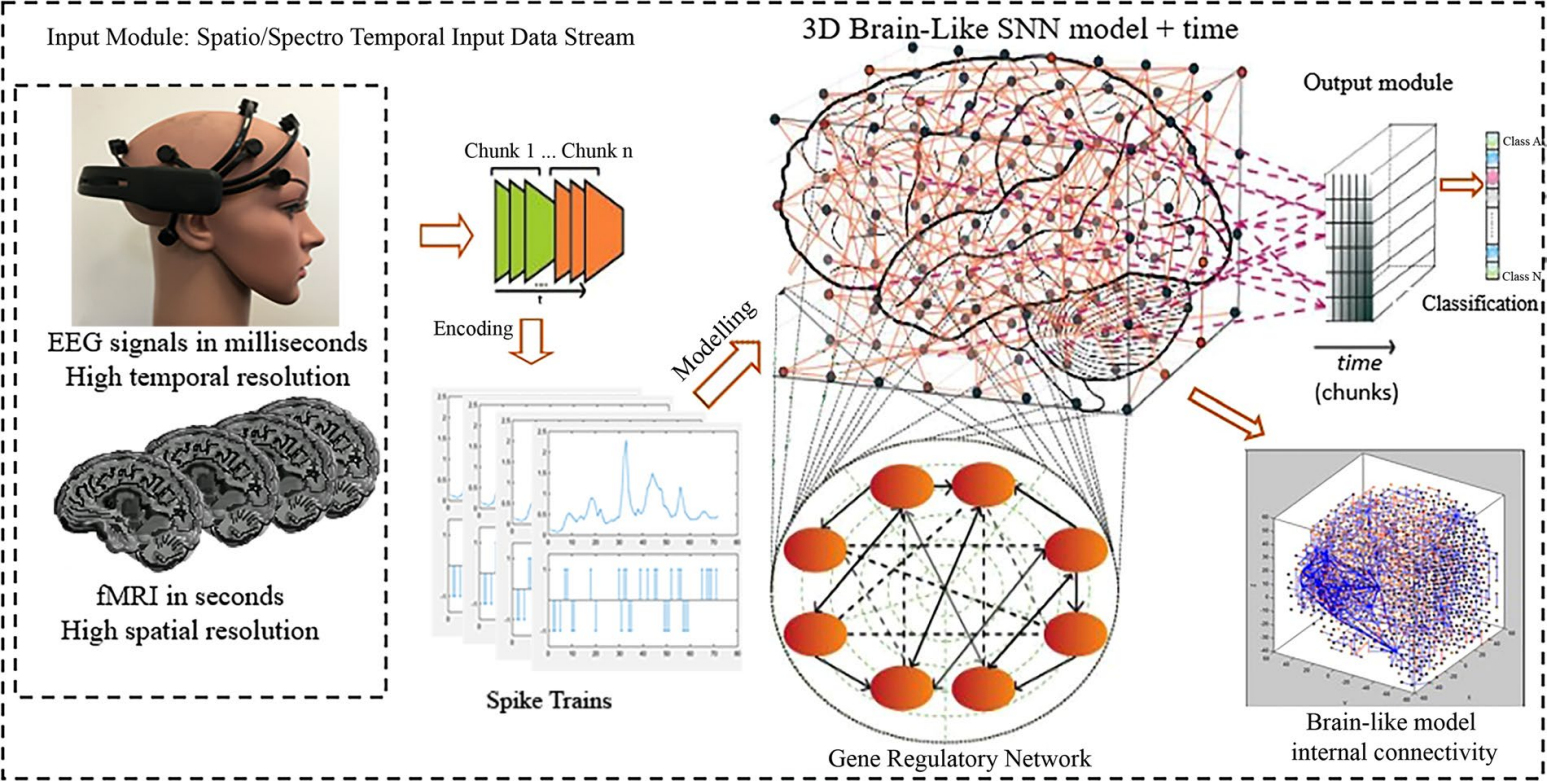
The SNN architecture, which contains several modules: input spike-time data encoding; a 3D SNN reservoir for unsupervised learning; a SNN classification/regression module using RO and STDP for supervised learning; gene regulatory network (GRN) as a system parameter optimisation model (optional and not used in the current paper). The Figure was drawn by authors Z.G. and M.D.

The proposed SNN-based methodology is based on the framework of evolving spiking neural networks, designed to learn from both temporal and spatial information^35^. The SNN architecture includes several functional modules (as shown in Fig. 6): an input-encoding module; a 3D SNN module for unsupervised training; an output classification/regression module for supervised training; an optimisation module; and a knowledge extraction and visualisation module^35,41^. These modules are described in the following sections.

#### Input Data Encoding and Mapping

The EEG signal from each electrode is translated into a spike train (as demonstrated in Supplementary Fig. 2), and spikes from a given electrode will enter to the SNN model at a particular location. Each electrode corresponds to a single unit (input neuron) in the SNN.

For a temporal signal *S*(*t*) over time *t* = 1, 2, …, *n*, the signal amplitude variation over time is denoted by *V*(*t*) where at baseline, *V*(1) = *S*(1). At the next time point *t*, if the upcoming signal amplitude *S*(*t*) is greater than *V*(*t*−1) + θ (sum with a threshold θ), then a positive spike is generated, whereas for a decreased signal, a negative spike is generated. The encoding of positive and negative spikes is defined as follows:

1 $$spike(t)=\{\begin{array}{ll}1\,then\,V(t)\leftarrow V(t-1)+\theta ; & if\,S(t)\ge V(t-1)+\theta \\ -1\,then\,V(t)\leftarrow V(t-1)-\theta ; & if\,S(t)\le V(t-1)+\theta \\ 0 & \,\,\,\,otherwise)\,\end{array}$$

As shown in Relation 1, the encoded spike sequences are in the form of binary events, in which −1 refers to a negative spike (the site of downward changes in signal values) and 1 is a positive spike (the site of upward changes). This method has been successfully used in dynamic vision sensors (DVS)^64^. Supplementary Fig. 2 shows an example of encoding EEG data recorded from the Cz channel into a sequence of positive and negative spikes using the TBR algorithm^35,65^. It shows that out of a total of 115 spikes generated, 58 were positive (indicated as +1) and 57 negative (indicated as −1). The spike trains are used for training the SNN model.

We defined a biologically plausible 3-dimensional SNN and initialised it with small-world connectivity^41,42^. Small-world structure is an organising principle in many natural systems, including networks of brain *neurons*, as both anatomical connections^66^ and synchronisation networks of cortical *neurons*^67^ exhibit small-world topology^68^. The neurons in the SNN reside at coordinates defined within the Talairach brain atlas, and neurons are connected probabilistically, such that neurons that are anatomically adjacent are very likely to be connected and those that are anatomically distant from one another are very unlikely to be connected. This constrains the space of potential interactions in a biologically plausible way. For instance, small-world networks are observed in several large-scale networks of brain *neurons*, such as the visual system^69^. The brain information processing is performed in both segregated and dispersed functional areas, as presented in^66–68,70^. Similarly, the small-world rule can include both short-distance connectivity (local clusters) within nearby *neurons* (similar to segregated information processing) and long-distance connectivity by linking the local clusters (similar to spread information processing). Using small-world rules in computational modelling has several advantages, as it supports high local and global efficiency in parallel processing, dynamic operation and rapid adaptation in network reconfiguration^71^. It also results in higher rates of information processing and learning than other techniques, such as random graphs^71^. These theoretical and empirical reasons led us to use small-world rules for the initialisation in the proposed SNN model. The SNN models are initialised using the small-world connectivity rule^41,42^ in which a probability of a neuron *i* to be connected to another neuron j depends on the distance between the two neurons, the larger the distance – the smaller the probability. In some cases, a radius is defined which represents the maximum distance of connections of one neuron to another in the 3D space of the SNN. The initial connections are assigned as small random weights, so that for example 80% of them are weighted by positive values while 20% of them are weighted by negative values. All the above parameters can be selected based on the task in hand. These initial connection weights are then adjusted by biologically plausible unsupervised learning rules which rely on the temporal dynamics and spiking activity triggered by input neurons as explained in the next section.

#### Unsupervised Learning in a 3D SNN Model

The known unsupervised spike-time-dependent plasticity (STDP) learning rule is used for learning in the SNN models proposed here. Through STDP learning, a connection *W*_*i, j*_ between *neurons* i and j is adapted according to the timing of their output spikes. If *neuron* i emits a spike earlier than j, then *W*_*i,j*_ will increase; otherwise, that would imply that neuron *j* is driving neuron *i* so *W*_*i*_, will decrease. STDP is described as follows:

2 $$F({\rm{\Delta }}t)=\{\begin{array}{ll}{A}_{+}\exp ({\rm{\Delta }}t/{\tau }_{+}) & if\,{\rm{\Delta }}t < 0\\ -{A}_{-}\exp (\,-{\rm{\Delta }}t/{\tau }_{-}) & if\,{\rm{\Delta }}t\ge 0\end{array}$$

*F*(Δ*t*) describes the adjustment of synaptic plasticity in respect to the pre-synaptic and post-synaptic spiking time in the interval of ${\rm{\Delta }}t={t}_{pre}-{t}_{post}$. The parameters A+ and A− are the maximum amounts for synaptic adjustment, which apply if Δ*t* is close to zero. The parameters *τ* + and *τ* − control the interval of pre- to post-synaptic spikes during which the weakening and the strengthening of the synaptic connection occur. During this learning procedure, the input neuron will accumulate spikes to the SNN model and, if neurons cross an activation threshold, they will also emit output spikes. That spike is sent out to all the units it is connected with, and what reaches each distal neuron is the spike scaled by the connection weight. That neuron will likewise accumulate activity as a function of receiving spikes and, after crossing some threshold, fire^45^. In such way, spikes are transferred between neurons and propagated to the SNN model. Therefore, the STDP rule captures ‘hidden’ spatio-temporal relations in the STBD stream, in the form of neuronal connections between spatially located *neurons* in the SNN model.

#### Supervised Learning and Classification using a SNN Classifier

At this step, a dynamic evolving SNN (deSNN)^35,72,73^, fully connected to all *neurons* in the 3D SNN, is used for classification/regression of the activated spiking patterns in the 3D SNN when input data are propagated through it. Other classifiers can also be employed^74^. The deSNN applies supervised learning in an output classifier layer using the class labels of the training samples. For each sample in the training set, one *neuron* is evolved in the output layer and linked to all the *neurons* in the already trained 3D SNN. The connection *W*_*ij*_ between *neuron* i from the 3D SNN and *neuron* j from the output layer is initialised by using a rank-order (RO) rule. The RO rule emphasises a higher priority for earlier spikes to an output *neuron*. Data with class labels are propagated through the trained 3D SNN and a supervised learning process is applied to train an output classifier. The potential *PSP*(*j*, *t*) of output *neuron* j at time t is defined using the following relation:

3 $$PSP(j,\,t)=\sum mo{d}^{order(i)}\,{W}_{ij}$$

where order (*i*) represents the order of the spike transmitted through *W*_*ij*_ and mod is a parameter. Therefore, the first spike that reached to the output *neuron* j from the 3D SNN model causes the highest increase in the corresponding connection weight. After the first spike has arrived, for the next spikes coming at time t from *neuron* i, the connection weight *W*_*ij*_ will rise by parameter drift; otherwise, *W*_*ij*_ will decrease by a drift value.

#### Parameter Optimisation

For model parameter optimisation, an exhaustive grid search method has been utilised to minimise the cross-validation classification error. The best classification accuracy has been obtained through searching over the main parameters (learning rate of STDP, neuron firing threshold, and deSNN classifier mode). Further explanation of these parameters is presented in^35,36^.

### Data availability

As supplementary material for the Nature Scientific Reports journal, we have made the EEG/ERP data available at the R&D systems of the Knowledge Engineering and Research Discovery Institute (KEDRI) website: https://kedri.aut.ac.nz/R-and-D-Systems/neuromarketing.

## Electronic supplementary material

Supplementary File

Video
