# Supplementary material for: Modelling Peri-Perceptual Brain Processes in a Deep Learning Spiking Neural Network Architecture
Source: Sci Rep. 2018 Jun 11;8:8912. doi: 10.1038/s41598-018-27169-8 (PMC5995966; doi:10.1038/s41598-018-27169-8)
Supplement: Supplementary file 1 — Supplementary File [file 41598_2018_27169_MOESM1_ESM.pdf]

Modelling Peri-Perceptual Brain Processes in a Deep Learning Spiking Neural Network Architecture

Authors: Zohreh Gholami Doborjeh [1*], Nikola Kasabov [1], Maryam Doborjeh [1*] and Alexander Sumich [2]

Affiliations:

[1]Knowledge Engineering and Discovery Research Institute, Auckland University of Technology, AUT Tower, 7th floor, 2 Wakefield street, Auckland 1010, New Zealand

[2]College of Business Law & Social Sciences, School of Social Sciences, Nottingham Trent University, Nottingham, United Kingdom

*__Correspondence to:__ Zohreh Gholami Doborjeh, zgholami@aut.ac.nz

Maryam Doborjeh, mgholami@aut.ac.nz

Supplementary Information, Figures, Tables and Movie's Caption

In this paper, we measured the role of peri-perceptual processes of the brain in consumers' preferences. Our hypothesis is that through learning in a SNN model, we can trace and understand how early the brain perceives familiar stimuli and the related spatio-temporal activities.

In the designed cognitive task, participants were instructed to respond to the target stimulus (logo of water) and skip the rest (non-target stimuli: familiar and unfamiliar logos). So, they were oblivious to the non-target stimuli. However, our analytical results showed that subjects' brain preferences have been also driven towards familiar and unfamiliar logos. Supplementary Fig. 3 illustrates that the neural connections are generated differently while the participants were dealing with different mental activities. When the participants were required to respond to the target logo, more neural connections were created within the SNN model (Supplementary Fig. 3b) in comparison to the non-target logos. Within the non-target logos, we found stronger SNN connectivity towards Familiar logos (200 ms after stimuli

presentation). Therefore, regardless of how the participants like or dislike the logos (familiar and unfamiliar), we could observe their brain peri-perceptual processes in response to familiar marketing stimuli.

To illustrate the differences between the SNN models of familiar versus unfamiliar logos learnt within the first 200ms, we visualised the differences between the connectivity of the two corresponding models (performed through subtraction) as shown in supplementary Fig. 4(c). This shows that at 200 ms after stimuli presentation, the most significant differences between familiar and unfamiliar stimuli have been observed around the O1, O2, P4, P3, F7 and F8 EEG channels as input variables to the two corresponding 3D SNN models.

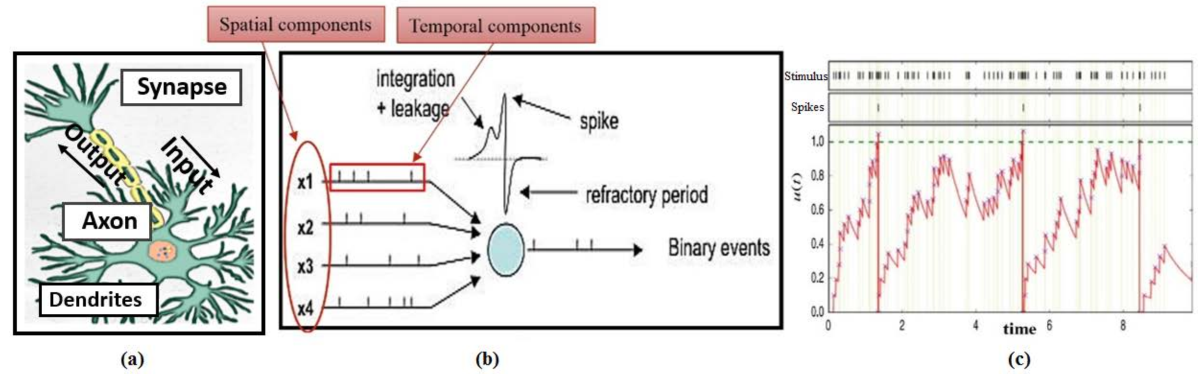

Figure 1. (a) Structure of a biological neuron which receives input information across axon terminals and processes it; (b) Picture of a simulated artificial spiking neuron with the model of Leaky-Integrate-Fire (LIF); (c) Functionality of LIF model using an input spike sequence (upper spike train), the output emitted spikes (middle spike train) and the changes of post-synaptic potential. The Figure (a, b and c) are drawn by authors Z. G and M. D.

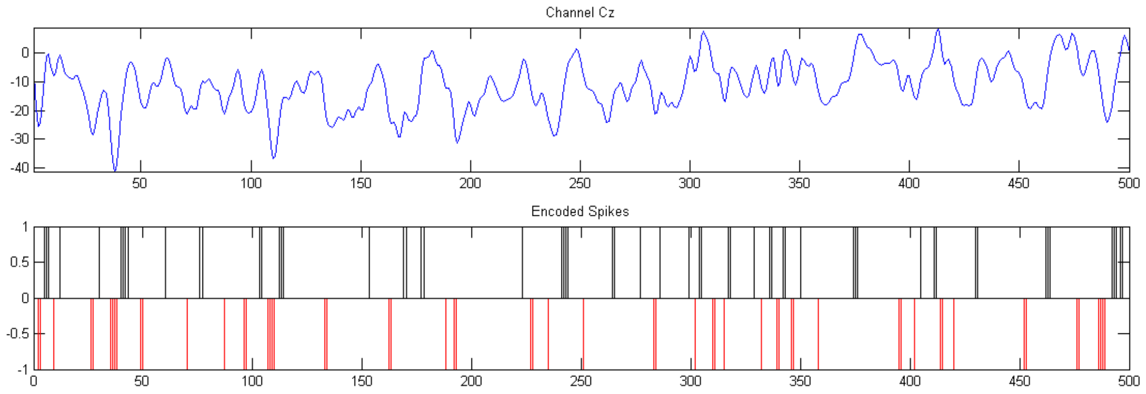

Figure 2. An example of encoding EEG data into sequence of positive (black) and negative spikes (red) using the TBR algorithm that is the format of the input data into the SNN architecture. The image shows 500 EEG data time points of electrode CZ from one subject.

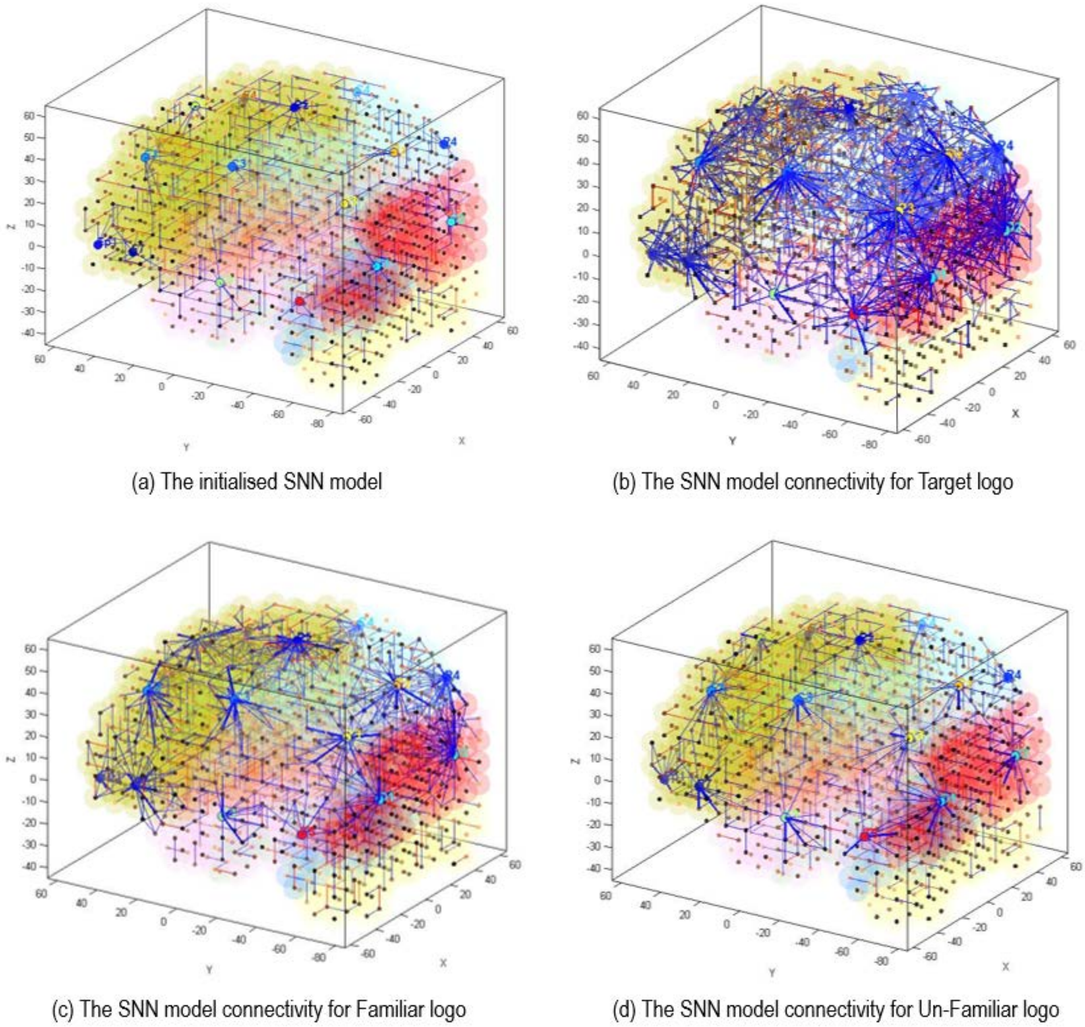

(a) The initialised SNN model

(b) The SNN model connectivity for Target logo

(c) The SNN model connectivity for Familiar logo

(d) The SNN model connectivity for Un-Familiar logo

Figure 3. Comparative visualisation of the SNN models which were trained by EEG data for (b) target logos; (c) non-target, familiar logos; (d) non-target, unfamiliar logos. The neuron connections were adapted after the STDP unsupervised learning by the EEG data of 200 milliseconds after stimuli presentation.

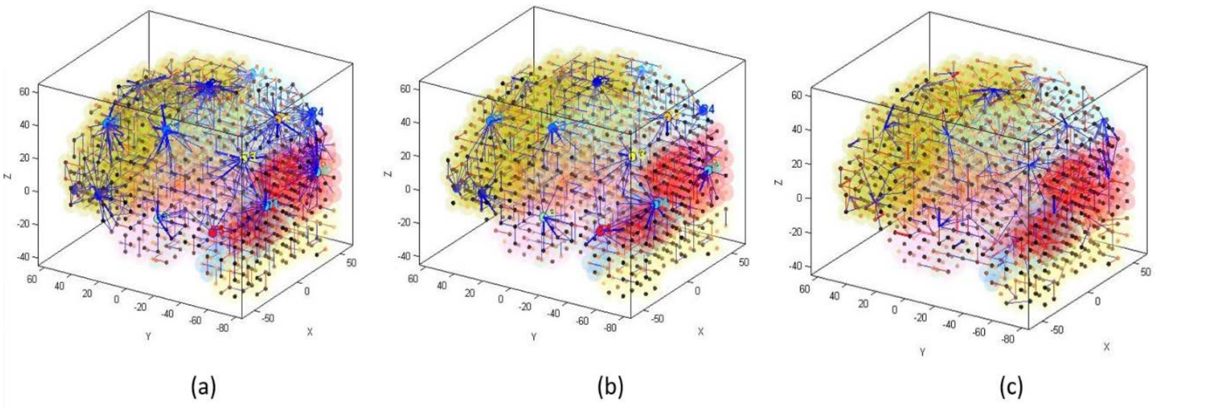

Figure 4. The connectivity of SNN models trained on 200 milliseconds of EEG data when stimuli (a) familiar logo stimuli are presented; (b) unfamiliar logo stimuli are presented; (c) the differences in connectivity between the SNN models (a) and (b) reflecting the spatio-temporal differences between perception of familiar versus unfamiliar logos.

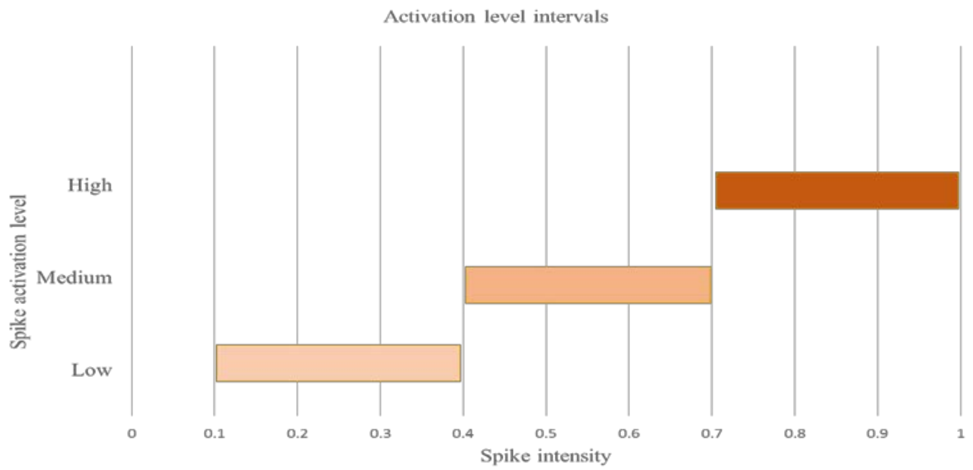

Figure 5. The levels of spike activation

Table 1. The mean amplitude of ERP components (P100 and P200) for familiar and unfamiliar logos in parietal lobe.

**ERP Components in Parietal Lobe**		**Mean amplitude of ERP components (P100)**		**Mean amplitude of ERP components (P200)**	
		**Familiar stimuli**	**Unfamiliar Stimuli**	**Familiar stimuli**	**Unfamiliar Stimuli**
**Electrode**	**P3**	3/77	2/02	4/33	4/25
	**P4**	4/02	1/89	4/38	4/20

Table 2. The mean amplitude of ERP components (N100 and P200) for familiar and unfamiliar logos in occipital lobe

ERP Components in Occipital Lobe		Mean amplitude of ERP components (N100)		Mean amplitude of ERP components (P200)	
		**Familiar stimuli**	**Unfamiliar Stimuli**	**Familiar stimuli**	**Unfamiliar Stimuli**
**Electrode**	**O1**	-3/96	-2/48	6/21	5/41
	**O2**	-5/80	-2/56	6/09	5/96

Table 3. Repeated-measures ANOVA for the ERP components P100 and P200 in Parietal lobe. Highlighted shows a significant main effect of the factor Familiarity and Electrode Site*Familiarity interaction at (P<0.05)

**Source**	**Mean amplitude of ERP components (P100)**			**Mean amplitude of ERP components (P200)**		
	**df**	**F**	**Sig**	**df**	**F**	**Sig**
Electrode	1	3/01	0/09	1	0/13	0/72
Familiarity	1	0/27	0/60	1	4/54	0/04
Electrode*Familiarity	3	0/28	0/32	3	4/61	0/01

Table 4. Repeated-measures ANOVA for the ERP components N100 and P200 in Occipital lobe. Highlighted shows significant main effects of Electrode Site, Familiarity and interaction between Electrode Site and Familiarity at (P<0.05).

**Source**	**Mean amplitude of ERP components (N100)**			**Mean amplitude of ERP components (P200)**		
	**df**	**F**	**Sig**	**df**	**F**	**Sig**
Electrode	3	11/45	0/01	1	0/14	0/70
Familiarity	1	51/3	0/01	1	0/80	0/38
Electrode*Familiarity	1	4/66	0/04	1	0/46	0/50

Table 5. The average of the connection weights between each input neuron (EEG channel) and a cluster of neighbouring neurons that are connected to it during the learning process with EEG epochs of 100 ms, 150 ms and 200 ms. The last column represents the average of the connection weights across all the EEG channels.

**Time frame**	**class**	**C3**	**Cz**	**C4**	**F7**	**F3**	**Fz**	**F4**	**F8**	**P3**	**Pz**	**P4**	**T3**	**T4**	**T5**	**T6**	**FP1**	**FP2**	**O1**	**O2**	**AVG**
**100 ms**	Familiar	0.23	0.18	0.26	0.52	0.68	0.73	0.09	0.32	0.22	0.29	0.37	0.11	0.98	0.73	0.38	0.15	0.11	0.05	0.36	0.35
	Unfamiliar	0.32	0.08	0.23	0.43	0.78	0.23	0.05	0.28	0.04	0.03	0.37	0.05	0.8	0.19	0.03	0.14	0.13	0.15	0.12	0.23
**150 ms**	Familiar	0.84	0.93	1.04	0.43	0.73	0.93	0.58	0.3	0.54	0.34	1.02	0.85	0.8	0.8	0.87	0.6	0.54	0.89	0.32	0.70
	Unfamiliar	0.3	0.27	0.84	0.07	0.66	0.2	0.11	0.19	0.13	0.06	0.78	0.76	0.03	0.74	0.99	0.06	0.32	0.04	0.79	0.38
**200 ms**	Familiar	1.3	1.33	0.76	1.03	1.05	0.65	0.32	1.05	1.01	0.97	1.12	1.12	1.19	1.06	1.04	0.89	1.03	1.32	1.02	1.01
	Unfamiliar	0.93	1.07	0.11	0.49	0.68	0.48	0.01	0.48	0.54	0.58	0.51	0.88	0.6	0.41	1.03	0.42	0.83	0.45	0.73	0.59

Table 6. The total number of spikes emitted by neurons at each time frame (Fig. 4 in the main manuscript) for EEG epochs of 50 ms, 100 ms, 150 ms, and 200 ms.

	**50**	**100**	**150**	**200**
**Familiar**	11	24	24	59
**Unfamiliar**	3	8	5	32
**Fam/Unfam**	3.7	3.00	4.6	1.84

Table 7. Intensity of the spikes emitted in clusters of neurons that surrounding EEG channels (input neurons) during the learning process with EEG epochs of 50 ms, 150 ms and 200 ms. The activation level is highlighted into 3 colours that are explained in Supplementary Figure 5.

**Time frame**	**class**	**C3**	**Cz**	**C4**	**F7**	**F3**	**Fz**	**F4**	**F8**	**P3**	**Pz**	**P4**	**T3**	**T4**	**T5**	**T6**	**FP1**	**FP2**	**O1**	**O2**
**50 ms**	Familiar	0	0	0	0	0	0	0	0	0	0	0	0	0.2		0	0	0		0.5
	Unfamiliar	0	0	0	0	0	0	0	0	0	0	0	0	0	0	0	0	0	0	0.3
**100 ms**	Familiar		0	0	0	0	0	0	0	0.2	0.5	0	0	0	0	0.6	0	0	0	0.6
	Unfamiliar	0	0	0	0	0	0	0	0	0	0	0	0	0	0	0.3	0	0	0.3	0.3
**150 ms**	Familiar	0	0.5		0.2	0	0	0	0	0.6	0.5	0.5	0	0	0.1	0	0.2	0	0	0
	Unfamiliar		0		0	0	0	0	0	0.2	0	0	0	0.2	0	0	0	0	0.1	0
**200 ms**	Familiar	0	0	0	0.3	0	0	0	0.6	0	0.7	0.8	0	0.9	0		0.2	0.9	0.2	0.8
	Unfamiliar	0	0	0	0	0	0	0		0		0.5	0	0.7	0	0.2	0	0.7	0.6	0.2

Supplementary Movie:

Neuromarketing data analytics based on SNN models for studying of mechanisms underpinning brain functions in response to marketing stimuli as revealed in three main phases: input data for encoding to sequence of spikes; a 3D model for spatially mapping of the brain data; an output for data analysis, classification and interpretation.
